# Treatment of sarcopenia and glucose intolerance through mitochondrial activation by 5-aminolevulinic acid

**DOI:** 10.1038/s41598-017-03917-0

**Published:** 2017-06-21

**Authors:** Chikako Fujii, Kazutoshi Miyashita, Masanori Mitsuishi, Masaaki Sato, Kentaro Fujii, Hiroyuki Inoue, Aika Hagiwara, Sho Endo, Asuka Uto, Masaki Ryuzaki, Motowo Nakajima, Tohru Tanaka, Masanori Tamaki, Ayako Muraki, Toshihide Kawai, Hiroshi Itoh

**Affiliations:** 1 Department of Endocrinology, Metabolism and Nephrology, Keio University School of Medicine, Tokyo, Japan; 2 SBI Pharmaceuticals Co., Ltd., Tokyo, Japan

## Abstract

Recently, sarcopenia has attracted attention as therapeutic target because it constitutes a risk factor for metabolic and cardiovascular diseases. We focused 5-aminolevulinic acid (ALA) which act as electron carriers in the mitochondrial electron transport system. The mice that received ALA for 8 weeks gained muscle strength and endurance, and exhibited increased muscle mass and mitochondrial amount. Administration of ALA to sarcopenia mice aged 100 weeks and chronic kidney disease (CKD) model mice also increased muscle mass and improved physical performance. Metabolome analysis revealed increased branched-chain amino acids (BCAAs) levels in the skeletal muscle of ALA-treated mice. Quantitative PCR analysis revealed decreased expression levels in branched-chain amino acid transaminases (BCATs) that degrade BCAAs and other muscle-degrading factors, and increased levels of mitochondria-activating factors. We also studied in cultured myocytes and obtained compatible results. ALA-treated mice tended to increase body weight, but reduced blood glucose level. These suggested that ALA treatment not only activated muscle mitochondria but also enhanced muscle mass through an increase in BCAAs contents, as to improve muscle strength, endurance and glucose tolerance in mice. In these ways, muscle mitochondrial activation with ALA is suggested to be useful for the treatment of sarcopenia and glucose intolerance.

## Introduction

As the world’s population is ageing, the World Health Organization (WHO) advocates the extension of healthy life expectancy as the new goal of medicine in an ageing society^1^. Healthy life expectancy is defined as an expected period of life requiring no nursing care and falls and fractures are major causes for shortening it^2^. Therefore, sarcopenia, which is usually defined as the status with decreases in muscle mass and physical performance, has drawn attention as a critical factor which affects healthy life expectancy. In addition, sarcopenia is recently shown to constitute the risk for obesity, diabetes and cardiovascular diseases; therefore, it is now attracting attention as a new target for medical treatment^3, 4^. Elucidating the mechanism for a decrease in muscle mass and establishing novel means to counteract it are recognized as significant goals for medical research in an ageing society.

Muscle mass begins decreasing in a person’s 20 s and decreases by 30% from the ages of 20 to 80^5^. Previous treatments for sarcopenia include exercise therapy involving resistance or aerobic exercise, diet therapy with a high-protein diet, and drug therapy mainly with anabolic steroids, with the common goal of increasing the muscle mass^6–10^. These treatments have a certain level of efficacy in increasing muscle mass and significantly improve the physical performance^11^. However, treatment to enhance muscle mass does not necessarily result in the reduction of cardiovascular diseases^12^. The function of skeletal muscle mitochondria, which is a factor as closely related to physical performance as muscle mass, markedly decreases with ageing, and the enzymatic activity of the mitochondrial electron transport system reduces by 50% from the ages of 20 to 80^13, 14^. The reduced muscle mitochondrial function has been reported to lead to a decrease in muscle mass and the development of obesity and diabetes^15, 16^. Therefore, we are focusing on the possibility that the mitochondrial dysfunction is a common cause of sarcopenia and metabolic diseases^17^.

5-aminolevulinic acid (ALA) is a mitochondria-activating substance, which is synthesized from glycine and succinyl-CoA by the action of ALA synthase in mitochondria. It serves as the first compound in the porphyrin synthesis pathway, which subsequently consists of an element of mitochondrial electron carriers, heme and cytochrome c. Cytochrome c is the key electron carrier in the mitochondrial electron transport system, and ALA supplementation with sodium ferrous citrate (SFC) has been shown to promote mitochondrial electron transport and increased ATP production^18^. In plants, ALA increases the production of chlorophyll, which is an essential molecule for photosynthesis and promotes growth^19^. Meanwhile, the effects of ALA in aged mice have not been fully studied yet.

The present study investigated the applicability of ALA for sarcopenia treatment. The effects of ALA on the skeletal muscle and on physical performance and glucose tolerance, which are closely related to muscle mitochondrial functions, were examined by use of ALA-treated mice. The effects of ALA on mitochondrial energy metabolism and the expression of related genes were also evaluated in the cultured myocytes.

## Material and Methods

### Materials and Animals

C57BL/6 mice were purchased from Charles River Laboratories (Tokyo, Japan). ALA and SFC were courtesy gifts from SBI Pharmaceuticals (Tokyo, Japan). C2C12 cultured myocytes were obtained from RIKEN BioResource Center (Tsukuba, Japan). C2C12 cells were grown to a nearly confluent number in Dulbecco’s Modified Eagle’s Medium (DMEM no. 11995; Gibco, Gaithersburg, MD) supplemented with 10% fetal bovine serum (FBS), and cells were differentiated into myocytes by incubation with 0.1% FBS for a few hours before the experiments. All animal experiments were approved by the local ethics committee (the Laboratory Animal Care and Use Committee of Keio University) and were conducted in accordance with the domestic law on the protection of laboratory animals, which is based on the Declaration of Helsinki.

### 5-aminolevulinic acid-treatment in mice

Male C57BL/6 mice were fed normal chow (10 kcal% fat, 20 kcal% protein, and 70 kcal% carbohydrate) and treated with 0.03% ALA (0.3 g/kg pellet) and SFC (5 mg/ml in drinking water) from 8 weeks of age. Control mice were also given SFC. The mice were measured for body weight from 8 to 16 weeks of age, and glucose tolerance and respiratory gases were examined at 16 weeks of age by the standard procedures. In some experiments, mice were fed high-fat diet (60 kcal% fat, 20 kcal% protein and 20 kcal% carbohydrate) or pair-feeding was performed. In addition to the conventional dose of ALA for animal experiments, a low dose treatment, which is equivalent to daily supplement tablets for human, was attempted. The low-dose ALA chow contained 0.003% ALA.

### Analysis of body composition, physical performance and metabolic parameters in 5-aminolevulinic acid-treated mice

The mice were examined for muscle strength, endurance, respiratory gases and glucose tolerance at 16 weeks of age, that is, after the mice were fed an ALA-containing diet or normal chow for 8 weeks. Muscle strength of the forelimbs was measured using a standard dynamometer for mice (MK-380M, Muromachi Kikai, Tokyo, Japan). Each mouse was placed on a metal mesh and pulled horizontally, and the power of traction when the mouse released the mesh was defined as the muscle strength. The method to estimate the muscle strength of rodents was originally developed by Dr. Meyer^20^. Measurements were repeated three times. Endurance was measured on a treadmill for mice (LE8170M, PanLab, Barcelona, Spain) according to the previously described protocol^21^. For the measurements of endurance, mice were forced to run on the motor-driven treadmill until they were completely exhausted, which was defined as the time point at which they remained on the electrical stimulating plate for more than 30 s. The treadmill was set at a constant 10% incline; the speed was 18 cm/s at the beginning and was increased by 3 cm/s every 2 min. The average time until exhaustion for wild-type mice fed with normal chow was 24 min (corresponding to 450 m of running). Oxygen utilization and the respiratory quotient were measured by a respiratory gas analyzer (ARCO-2000 mass spectrometer, ARCO system, Kashiwa, Japan). Respiratory gas analysis was performed at 16 weeks of age. To perform the intra-peritoneal glucose tolerance test (ipGTT), the mice were forced to fast for 16 h overnight and then intra-peritoneally injected with glucose at a dose of 1 g glucose/kg body weight. In high-fat diet-fed mice and db/db obese mice, they were intra-peritoneally injected with glucose at a dose of 0.5 g glucose/kg body weight. Blood glucose levels were measured before glucose injection and at 15, 30, 60 and 120 min after the glucose injection.

To obtain cross-sectional images of the mice and to estimate the ratio of the fat mass and the muscle mass at the total body level, an analysis using micro-computed tomography (micro-CT) was performed (R_mCT2, RIGAKU, Tokyo, Japan) which produces images with a voxel size of 118 μm^3^ cube. The sequential images were obtained from whole body of the mice for the length of 6 cm ranging from the neck to the root of the tail (512 images/ a mouse). Twenty images at the pitch of 0.3 cm were extracted from the sequential images of the mice and the areas of fat, muscle and body in each image were measured and multiplied by the thickness (0.3 cm). The values were summed up for the 20 images and regarded as the estimated volume^22, 23^. To quantify the ratio of fat mass and muscle mass at the total body level, the estimated volume of each portion was divided by the total body volume.

The mice were sacrificed after each mouse was examined for body composition, physical performance and metabolic parameters, and various tissues were harvested. We measured the muscle weight (quadriceps and gastrocnemius) and epididymal fat. The tissue samples from the quadriceps and gastrocnemius were used for histological analysis. The electrical microscopic analysis of the mitochondria of quadriceps muscle was performed by the standard method. A part of these tissues was used for quantitative PCR and western blotting analysis.

### Estimation of gene expression and protein levels by quantitative PCR and western blotting

Estimation of the gene expressions and protein levels of ALA-treated mice was performed by quantitative PCR and western blotting analysis, respectively by the standard method. Total RNA was extracted with an RNeasy Mini Kit (Qiagen), and reverse transcription was performed with an ExScript RT reagent kit (Takara Bio, Otsu, Japan). Gene expression levels were determined by quantitative PCR reactions (ABI 7500) in the presence of SYBR Premix ExTaq (Takara Bio) fluorescent dye. The genes examined by real-time PCR were as follows: the mitochondria-related genes; peroxisome proliferator-activated receptor gamma coactivator 1α (PGC1α), uncoupling protein 3 (UCP3), cytochrome c oxidase subunit IV (COXIV), ATP synthase (ATPsyn); the muscle protein degradation genes; atrogin1 and muscle RING-finger protein-1 (MuRF1); the branched chain aminotransferase genes 1, 2 (BCAT1, 2). The relative quantity of mRNA was normalized to that of the internal control, the 18 S gene. The mitochondrial amount in tissues isolated from each of the gastrocnemius, quadriceps, kidney, liver, heart and white adipose tissue (WAT) was measured as the level of mitochondrial DNA copy number. The number was estimated by the ratio of mitochondria DNA (16 S rRNA) to nuclear DNA (hexokinase 2 gene, intron 9).

Western blotting was performed by the standard methods to evaluate the protein levels and phosphorylation status of ribosomal protein S6 kinase1 (S6K1 or p70 S6 kinase)^24^. Total protein extracts (10 μg) from quadriceps were separated by polyacrylamide gel electrophoresis, transferred to nitrocellulose membranes using a dry blotting system (Trans-Blot Turbo; Bio-Rad, Hercules, CA) and incubated with antibodies. Immuno-labelled proteins were detected by using a chemiluminescence kit (ECL Plus; GE Healthcare, Piscataway, NJ) and a lumino-image analyzer (LAS-4000; FujiFilm, Tokyo, Japan). The density of the blot was estimated by imaging software (MultiGauge; FujiFilm). The primary antibodies used for western blotting were as follows: S6K1 (1:1000; #9202, Cell Signaling, MA) and phosphorylated S6K1 (P-S6K1, 1:1000; #9205, Cell Signaling).

### Metabolome analysis in the 5-aminolevulinic acid-treated mice

ALA has been shown to activate mitochondria by promoting the mitochondrial electron transport system. However, the precise mechanism by which ALA affects muscle mass remains unknown. Thus, we conducted metabolome analysis of the quadriceps muscle in ALA-treated mice using capillary electrophoresis-mass spectrometry (CE-TOFMS, Human Metabolome Technologies, Tsuruoka, Japan). Representative 116 metabolites, including the intermediates of the glycolytic system, TCA cycle, energy-associated metabolites and amino acids were analyzed in the quadriceps of ALA-treated mice. For details, the website available is http://humanmetabolome.com/.

### Experiments using C2C12 cultured myocytes

C2C12 cells were grown and differentiated in 24-well assay plates and treated with ALA or antimycin A or a combination of them (ALA, 10^−7^ mol/l, antimycin A, 10^−8^ mol/l, unless otherwise indicated) for 48 hours. Antimycin A is an inhibitor of cytochrome c reductase, which is the complex III in the mitochondrial electron transport chain. The mitochondrial and nuclear density of C2C12 cells were determined by using fluorescent dyes, Mito Tracker Green FM and Mito SOX Red (Molecular Proves, Eugene, OR), as described previously. C2C12 cells (sparse dissemination at 10^8^ cells/cm^2^) were visualized by means of a confocal microscope (LSM510; Carl Zeiss, Oberkochen, Germany) with staining by the fluorescent probes. Images were acquired at ×200 magnification. The C2C12 cells were examined for oxygen consumption rate, mitochondrial amount and gene expressions. The oxygen consumption rate of C2C12 cells was measured by an extra cellular flux analyzer (XF-24, Seahorse Bioscience, North Billerica, MA). Mitochondrial amount was determined by the DNA copy number. Gene expression levels were determined by quantitative PCR reactions (ABI 7500, Applied Biosystems, Foster City, CA) in the presence of SYBR Premix Ex Taq (Takara Bio) fluorescent dye.

### Statistical analysis

All data were expressed as mean ± standard error. Comparison of means between the two groups was performed by the Student’s t-test. When more than two groups were compared, analysis of variance was used to evaluate significant differences among groups, and if significant differences were confirmed, each difference was further examined by Fisher’s protected least significant difference method. P < 0.05 was considered to be statistically significant.

## Results

### 5-aminolevulinic acid improves physical performance by activating mitochondria and increasing muscle mass

**Figure 1:**
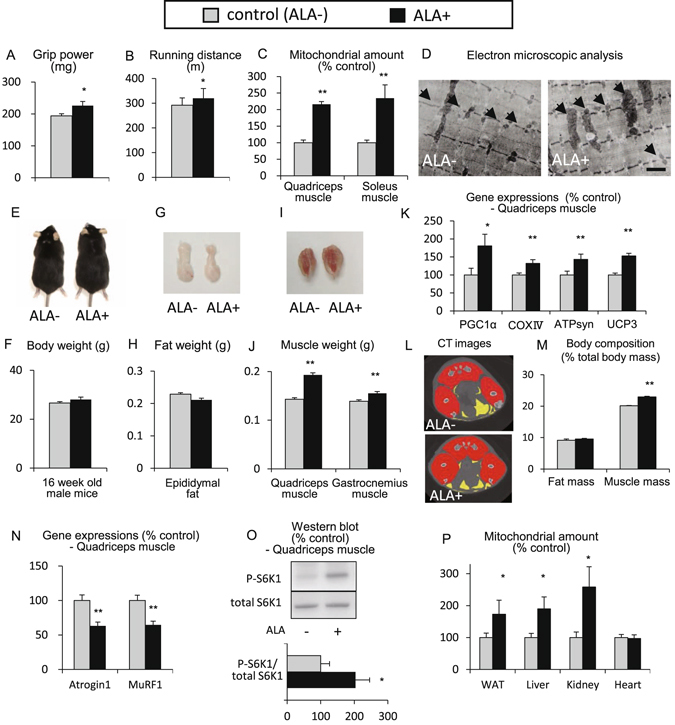
5-aminolevulinic acid improves physical performance by activating mitochondria and increasing muscle mass. To investigate the effects of ALA on muscle mitochondria and physical performance, C57BL/6 mice were fed with an ALA-containing diet (0.03% ALA) for 8 weeks from 8 weeks of age. Control mice (ALA−) and ALA-treated mice (ALA+) were examined. (**A**) Grip power at 16 weeks (16 W) of age as an index of muscle strength, (**B**) Running distance at 16 W as an index of endurance, (**C**) Mitochondrial amount in the quadriceps muscle and soleus muscle, estimated from mitochondrial DNA copy number, (**D**) Electron microscopic analysis of the quadriceps muscle. Scale bar = 1 µm, (**E**,**G** and **I**) Macroscopic view of the mice, epididymal fat and gastrocnemius muscle with soleus muscle, (**F**,**H** and **J**) The body weight, weight of the epididymal fat, and weight of the quadriceps and gastrocnemius muscle, (**K**) expression of mitochondria-related genes estimated by quantitative PCR analysis (PGC1α, COXIV, ATPsyn and UCP3), (**L**) Micro-CT images at the level of the maximal thigh circumference of the mice. The red part is the muscle, the yellow part is the fat, (**M**) The body composition of the ALA-treated mice at the total body level. The ratios of fat mass and muscle mass to total body volume estimated from the micro-CT images are shown; N = 4 in each group, (**N**) expression of muscle degradation-related genes estimated by quantitative PCR analysis (Atrogin1, MuRF1), (**O**) Western blots of P-S6K1 and total S6K1 (upper) and densitometry of the ratio of P-S6K1/total S6K1, as an index of S6K1 activity (lower). Full-length blots are included in the Supplementary Information, (**P**) Mitochondrial amount in various tissues (WAT, liver, kidney and heart). N = 8 in each group unless otherwise indicated. *P < 0.05, **P < 0.01 versus control mice. ALA, 5-aminolevlinic acid; PGC1α, peroxisome proliferator-activated receptor- coactivator 1; COXIV, cytochrome c oxidase subunit IV; ATPsyn, adenosine triphosphate synthase; UCP3, uncoupling protein 3; CT, computed tomography; Murf1, muscle specific ring finger protein 1; P-S6K1, phosphorylated S6 kinase1; WAT, white adipose tissue.

To investigate the effects of ALA on muscle mitochondria and physical performance, mice were fed with an ALA-containing diet for 8 weeks from 8 weeks of age. Muscle strength, endurance and mitochondria of ALA-treated mice were evaluated. The muscle strength and endurance were significantly increased by ALA treatment (Fig. 1A,B). The mitochondrial DNA copy number, which reflects the amount of mitochondria, was significantly increased in both white muscles (quadriceps muscle) and red muscles (soleus muscle) (Fig. 1C). Electron microscopic observation revealed an increased number of enlarged mitochondria in the quadriceps muscle of ALA-treated mice (Fig. 1D). ALA-treated mice tended to gain body weight but the increase was not always of significance (Fig. 1E,F). ALA administration induced a significant increase in muscle mass with no significant change in white adipose tissue weight (Fig. 1G–J). PCR-analysis of gene expression that activates muscle mitochondria showed that ALA administration increased the expression levels of PGC1α, COX4i1, ATPsyn and UCP3 in the skeletal muscle (Fig. 1K). Micro-CT analysis revealed that ALA administration induced a change in the body composition of the mice fed on the normal chow (Fig. 1L). The ratio of fat mass at the total body level, which was estimated from the micro-CT images, was unchanged after ALA-treatment, however, that of muscle mass was significantly increased (Fig. 1M). Expression levels of genes of muscle-degrading factors atrogin1 and MuRF1 were reduced (Fig. 1N). The activity of the muscle mass regulating factor, S6K1, which was assessed by the phosphorylation ratio (P-S6K1/ total S6K1) in western blotting, was increased in the quadriceps muscle of ALA-treated mice (Fig. 1O). The increase in mitochondrial amount by ALA treatment was significant in the WAT, liver and kidney but not in the heart (Fig. 1P). These results indicate that ALA, a precursor of mitochondrial electron carriers, improves physical performance in mice by activating skeletal muscle mitochondria and increasing muscle mass.

### 5-aminolevulinic acid improves the physical performance of aged mice with sarcopenia and chronic kidney disease model mice with decreased physical performance

ALA was administered for 8 weeks to 100-week-old mice with sarcopenia and 20-week-old 5/6 nephrectomized (5/6Nx) mice with decreased physical performance. Aged mice at 100 weeks of age exhibited age-associated decreases in muscle mass and mitochondria. They exhibited decreases in muscle strength, exercise endurance and glucose tolerance. In 5/6Nx chronic kidney disease (CKD) model mice at 20 weeks of age, in which nephrectomy was performed from 7 to 8 weeks of age, a decreased level of the mitochondrial amount was noticeable, but no significant decrease in muscle mass was observed^25^. They showed decreases in exercise endurance and glucose tolerance, even though muscle mass and power was maintained.

**Figure 2:**
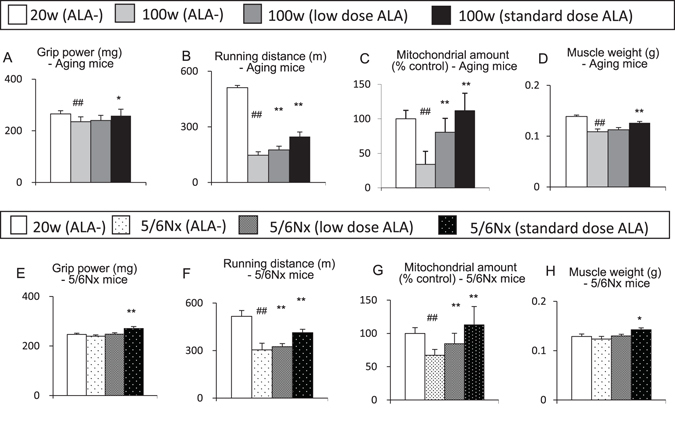
5-aminolevulinic acid improves the physical performance of ageing mice with sarcopenia and chronic kidney disease model mice with decreased physical performance. (**A–D**) Ageing mice (**A**) Grip power, (**B**) Running distance, (**C**) Mitochondrial amount and (**D**) Muscle weight of the gastrocnemius muscle), (**E–H**) 5/6Nx mice (**E**) Grip power, (**F**) Running distance, (**G**) Mitochondrial amount, (**H**) Muscle weight of the gastrocnemius muscle). N = 8 in each group. *P < 0.05, **P < 0.01 versus aged mice without ALA-treatment or 5/6 Nx mice without ALA treatment group. ^#^P < 0.05, ^##^P < 0.01 versus the control group. ALA, 5-aminolevlinic acid; 5/6Nx, 5/6 nephrectomized.

ALA was administered to the aged mice or 5/6Nx mice at a standard dose in animal studies (0.03% ALA, or standard dose ALA) or a low dose used as a human supplement (0.003% ALA, or low dose ALA). The muscle strength improved in aged mice that received ALA at the standard dose for 8 weeks (Fig. 2A). The endurance improved with the low dose and standard dose (Fig. 2B). The mitochondrial amount in the skeletal muscle was increased with the low dose and standard dose (Fig. 2C). The muscle mass was increased with the standard dose (Fig. 2D). A similar experiment was conducted in 5/6Nx mice. ALA administered to 5/6Nx mice significantly improved the muscle strength with the standard dose (Fig. 2E). The endurance improved with the low dose and standard dose (Fig. 2F). The amount of muscle mitochondria was increased with the low dose and standard dose (Fig. 2G). The muscle mass improved with the standard dose (Fig. 2H). These results suggest that ALA improves physical performance not only by activating mitochondria but also by increasing muscle mass in aged mice with sarcopenia and CKD mice with decreased physical performance.

### Branched-chain amino acids increase in the skeletal muscle of 5-aminolevulinic acid-treated mice

We performed metabolome analysis of each metabolite in the glycolytic system, TCA cycle, adenine nucleotides and nicotinamide derivatives, which are involved in the energy metabolism and the concentrations of individual amino acids in the quadriceps of ALA-treated mice. The results of the mean values for representative metabolites are shown in Table 1.

**Table 1:** Metabolome analysis of the metabolites in the quadriceps of ALA-treated mice.

Metabolites (nmol/g)			ALA−	ALA+	*p*-value
Glycolytic system	Glucose 6-phosphate		620 ± 281	292 ± 64	0.175
	Glucose 1-phosphate		51 ± 28	20 ± 4.4	0.186
	2-Phosphoglyceric acid		6.4 ± 2.2	3.9 ± 1.7	0.192
	Glycerol 3-phosphate		587 ± 102	916 ± 107	0.015*
TCA cycle	Succinic acid		182 ± 26	95 ± 13	0.014*
Energy-associated metabolites	NAD+		439 ± 9.5	400 ± 5.0	0.008**
	GTP		116 ± 6.8	94 ± 15	0.108
Amino acid metabolites	BCAA	Ile	15 ± 8.1	36 ± 6.7	0.026*
		Leu	67 ± 7.2	86 ± 12	0.090
		Val	54 ± 23	211 ± 26	0.022*
	Asp		131 ± 31	171 ± 4.9	0.149
	Glu		1,833 ± 64	1,492 ± 17	0.008**
	Lys		142 ± 43	70 ± 0.9	0.100
	Met		50 ± 2.9	46 ± 3.3	0.132
	His		125 ± 21	100 ± 6.2	0.160
	Arg		65 ± 9.4	46 ± 1.9	0.065

The quadriceps of control mice (ALA−) and ALA-treated mice (ALA + ) were examined. The representative metabolites that had a p-value < 0.2 of the difference between ALA- and ALA + are shown.

N = 3 in each group. *P < 0.05, **P < 0.01 versus control groups. ALA, 5-aminolevlinic acid; TCA cycle, tricarboxylic acid; NAD, nicotinamide adenine dinucleotide; GTP, guanosine triphosphate; BCAA, branched chain amino acid; Ile, isoleucine; Leu, leucine; Val, valine; Asp, aspartic acid; Glu, glutamic acid; Lys, lysine; Met, methionine; His, histidine; Arg, arginine.

**Figure 3:**
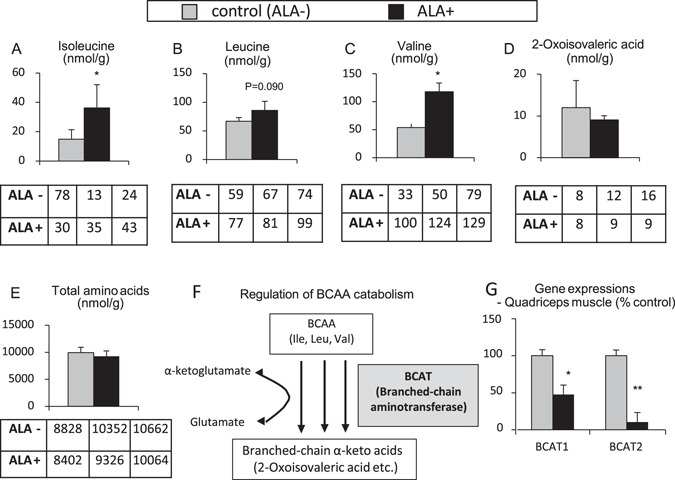
Branched-chain amino acids increase in the skeletal muscle of 5-aminolevulinic acid-treated mice. (**A**–**E**) Metabolome analysis in the quadriceps muscle (**A**) Isoleucine, (**B**) Leucine, (**C**) Valine, (**D**) 2-Oxoisovaleric acid, (**E**) Total amino acids), (**F**) Schematic representation of the regulation of BCAA catabolism by BCAT, (**G**) Gene expressions of BCATs estimated by quantitative PCR analysis (BCAT1, BCAT2). N = 3 in each group. *P < 0.05, **P < 0.01 versus control group. ALA, 5-aminolevlinic acid; BCAA, branched chain amino acid; BCAT, branched-chain amino acid aminotransferase.

ALA administration in mice induced many metabolic changes in the skeletal muscle. In particular, BCAAs, which are known to increase muscle mass by activating the TORC1/ S6K1 system, were increased in the quadriceps muscle of ALA-treated mice. Isoleucine and valine increased significantly, and leucine also demonstrated an upward trend (Fig. 3A–C). Oxoisovaleric acid, a degradation product of valine, tended to decrease, although the BCAAs increased (Fig. 3D). Degradation products of isoleucine and leucine were not successfully quantified. Total amino acids also tended to decrease (Fig. 3E). Because BCAAs are essential amino acids and enhanced biosynthesis of BCAA is unlikely, the increase in BCAAs should be due to a suppression of the degradation (Fig. 3F). In fact, the expression levels of BCATs (BCAT1 and BCAT2), enzymes responsible for the degradation of BCAAs, were markedly decreased in the quadriceps muscle of ALA-treated mice (Fig. 3G).

### Promotion of mitochondrial electron transport by 5-aminolevulinic acid affects the expression of genes regulating mitochondrial activity and muscle mass in cultured myocytes

The effects of mitochondrial activation with ALA on gene expression were investigated using cultured myocytes, C2C12. C2C12 cells were cultured in a 24-well plate. ALA and antimycin A, which binds to cytochrome c reductase (the complex III in the mitochondrial electron transport chain) and inhibits mitochondrial electron transport and ATP production, were added to the cultured cells, and their effects on the mitochondrial activity and the expression of genes regulating skeletal muscle mass were investigated.

**Figure 4:**
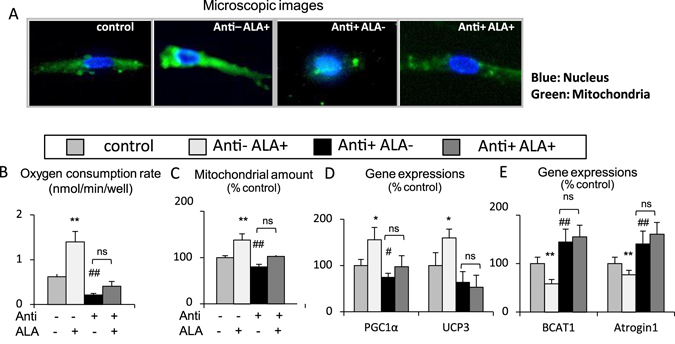
Promotion of mitochondrial electron transport by 5-aminolevulinic acid affects the expression of genes regulating mitochondrial activity and muscle mass in cultured myocytes. (**A**) Microscopic images of C2C12 cells (blue; nucleus, green; mitochondria), (**B**) Oxygen consumption rate (OCR), (**C**) Mitochondrial amount, (**D**) Expressions of mitochondria-related genes estimated by quantitative PCR analysis (PGC1α, UCP3), (**E**) Gene expressions of BCAT1 and the Atrogin1 estimated by quantitative PCR analysis. N = 5 independent experiments. *P < 0.05, **P < 0.01 versus ALA plus antimycin A. ^#^P < 0.05. ^##^P < 0.01 versus control group. ns: not significant between antimycin A versus ALA plus antimycin A. ALA, 5-aminolevlinic acid; PGC1α, peroxisome proliferator-activated receptor-γ coactivator 1α; UCP3, uncoupling protein-3; BCAT, branched-chain amino acid aminotransferase.

Observations with a fluorescence microscope revealed that fluorescent dye-labelled mitochondria were significantly increased in the ALA-added group and decreased in the antimycin A-added group (Fig. 4A). The oxygen consumption rate of the cells measured with a metabolic flux analyzer significantly increased by addition of ALA and this increase was completely abrogated by addition of antimycin A. Under the treatment with antimycin A, the oxygen consumption rate showed no significant difference by addition of ALA (Fig. 4B). Similarly, the mitochondrial DNA copy number, which reflects the mitochondrial amount, was significantly increased by ALA, and the increase was suppressed by antimycin A. However, under the treatment with antimycin A, the mitochondrial amount showed no significant increase by ALA (Fig. 4C). The expression levels of genes controlling the mitochondrial activity (PCG1α, a key regulator of mitochondrial biogenesis, and UCP3) or muscle mass (BCAT1 and atrogin1) were measured by quantitative PCR. The expression levels of PCG1α and UCP3 were increased by ALA, and those of BCAT1 and atrogin1 were decreased by ALA. Conversely, the expression levels of PCG1α and UCP3 were decreased by antimycin A, and those of BCAT1 and atrogin1 were increased by antimycin A (Fig. 4D,E). Under the treatment with antimycin A, the expression levels of PCG1α, UCP3, BCAT1 and atrogin1 showed no significant changes by ALA (Fig. 4D,E). These findings indicated that the degree of mitochondrial electron transport, which was inhibited by antimycin A, affected not only oxygen consumption in the cultured myocytes but also mitochondrial amount and expression of genes controlling the mitochondrial activity and muscle mass. Therefore, the effects of ALA on muscle mitochondria and muscle mass, which were observed in mice, were implied to be mediated by the promotion of mitochondrial electron transport.

### 5-aminolevulinic acid improves glucose tolerance without reducing body weight

**Figure 5:**
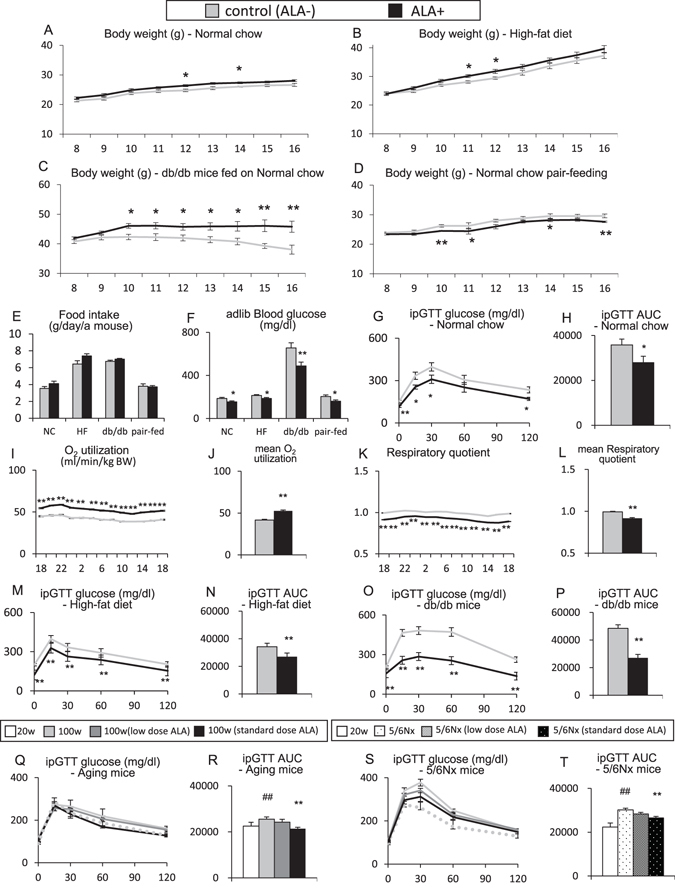
5-aminolevulinic acid improves glucose tolerance without reducing body weight. (**A**–**D**) Body weight (**A**) normal chow at 8–16 weeks (8–16); N = 10 in each group, (**B**) High-fat diet; N = 10 in each group, (**C**) db/db obese mice on normal chow; N = 5 in each group, (**D**) Normal chow pair-fed mice; N = 10 in each group), (**E**) Food intake (average daily intake per mouse), (**F**) Adlib blood glucose at 16 weeks of age (16 W), (**G**) ipGTT in ALA-treated mice with normal chow, (**H**) Area under the curve (AUC) of ipGTT, (**I**) Oxygen utilization of ALA-treated mice with normal chow (16 W); N = 6 in each group, (**J**) Mean oxygen utilization, (**K**) Respiratory quotient of ALA-treated mice with normal chow (16 W); N = 6 in each group, (**L**) Mean respiratory quotient, (**M**) ipGTT in ALA-treated mice with high-fat diet; N = 5 in each group, (**N**) ipGTT AUC (high-fat diet), (**O**) ipGTT in ALA-treated db/db obese mice; N = 5 in each group, (**P**) ipGTT AUC (db/db mice), (**Q**) ipGTT in ALA-treated mice with normal chow 20-week-old mice (20 W), control 100-week-old mice (100 W), low dose ALA-treated aged mice, standard dose ALA-treated aged mice; N = 8 in each group, (**R**) ipGTT AUC (aged mice), (**S**) ipGTT in ALA-treated mice with normal chow; 20-week-old mice (20 W), control 5/6Nx mice, low dose ALA-treated 5/6Nx mice, standard dose ALA-treated 5/6Nx mice, (**T**) ipGTT AUC (5/6Nx mice); N = 8 in each group. *P < 0.05, **P < 0.01 versus aged mice without ALA-treatment or 5/6Nx mice without ALA treatment group. ^#^P < 0.05, ^##^P < 0.01 versus the control group. ALA, 5-aminolevulinic acid; ipGTT, intra-peritoneal glucose tolerance test; AUC, area under the curve; 5/6Nx, 5/6 nephrectomized.

Eight-week-old C57BL/6 mice were fed with an ALA-containing diet for 8 weeks, and body weight changes in normal chow-fed mice, high-fat diet-fed mice, db/db diabetes model obese mice, and mice under food intake control by pair feeding were followed. The results of food intake, casual blood glucose, glucose tolerance testing and respiratory gas analysis at 16 weeks of age, which is 8 weeks after beginning ALA administration, were examined. In normal chow-fed mice and high-fat diet-fed mice, the body weight tended to increase after administration of ALA (Fig. 5A,B). In db/db mice, the body weight significantly increased after administration of ALA (Fig. 5C). In mice that underwent pair feeding to match food intake between the ALA-treated group and untreated group, the body weight significantly decreased after ALA administration (Fig. 5D,E). The food consumption tended to increase after initiation of ALA administration in all but pair-fed groups (Fig. 5E). Casual blood glucose levels significantly decreased in all ALA-treated mice (Fig. 5F). In the ipGTT of mice fed on the normal chow, the blood glucose levels after glucose loading significantly decreased in ALA-treated mice (Fig. 5G,H). The respiratory gas analysis showed that oxygen consumption significantly increased, and respiratory quotient significantly decreased in ALA-treated mice fed on normal chow (Fig. 5I–L). In the ipGTT of mice fed on the high-fat diet, the blood glucose levels after glucose loading significantly decreased in the ALA-treated mice (Fig. 5M,N) and db/db obese mice (Fig. 5O,P). The body composition of the obese mice was examined with the micro-CT analysis which was also used to examine the mice fed on normal chow. The muscle mass ratio at the total body level was significantly increased by ALA-treatment in mice fed on high-fat diet (17.5 ± 0.7 vs 20.9 ± 0.3%, P < 0.01, N = 4 in each group) and db/db obese mice (14.0 ± 0.3 vs 15.3 ± 0.2%, P < 0.01, N = 4 in each group). The fat mass ratio tended to decrease in these obese mice (30.5 ± 0.7 vs 28.4 ± 0.8% in mice fed on high-fat diet, P = 0.11; 50.8 ± 1.0 vs 47.1 ± 0.7% in db/db mice, P < 0.01, N = 4 in each group). Similarly, ipGTT was performed in aged mice and 5/6Nx mice with ALA-treatment for 8 weeks. In aged mice, the blood glucose levels after glucose loading significantly decreased with standard dose ALA (Fig. 5Q,R). In 5/6Nx mice, the blood glucose levels also significantly decreased with standard dose ALA (Fig. 5S,T).

Thus, in the normal chow-fed mice, high-fat diet-fed mice or db/db mice, casual blood glucose levels and those after glucose loading were reduced by ALA-treatment, while these animals tended to gain weight. With pair-feeding to match the food intake, ALA-treated mice significantly lost weight. ALA-induced improvements in glucose tolerance were observed not only in young mice but also in the aged mice and 5/6 nephrectomized mice.

## Discussion

In this study, we investigated the applicability of the mitochondrial activating substance, ALA, for the treatment of sarcopenia. ALA-treated mice exhibited an increased muscle mass as well as activated muscle mitochondria, and improved muscle strength, endurance and glucose tolerance. These results suggest that ALA supplementation is useful for sarcopenia treatment. In the muscles of ALA-treated mice, the expression levels of mitochondrial activating factors, such as PGC1α, were elevated, and the activity of a muscle synthesizing factor, target of rapamycin complex 1 (TORC1), was enhanced. The metabolome analysis revealed increased levels of BCAAs, which activate TORC1. The expression of enzymes responsible for BCAA degradation, BCATs, exhibited a decrease by ALA-treatment. In cultured myocytes, the effects of ALA on increasing PGC1α expression and decreasing BCATs expression were eliminated by inhibiting the mitochondrial electron transport system with antimycin A. From these results, the mitochondrial activation with ALA appears to increase muscle mass through an enhancement in BCAAs contents due to decreased BCATs expression in the skeletal muscle.

ALA has been investigated for therapeutic applications to pathological conditions based on mitochondrial dysfunction. In rats that had received a renal mitochondrial toxin, cisplatin, ALA increased the gene expression of molecules constituting the mitochondrial electron transport system, such as COXIV and ATPsyn, and reduced the cisplatin toxicity and renal tubular necrosis^26^. Despite the function of muscle mitochondria being closely related to the physical performance, there is very limited knowledge regarding the effects of ALA on the skeletal muscle. Although the efficacy of ALA on physical performance of elderly women has recently been reported by Masuki *et al*.^27^, the usefulness of ALA for sarcopenia treatment has not been investigated yet. To examine the therapeutic significance of ALA, we thus investigated physical performance and glucose tolerance of aged mice with sarcopenia and 5/6Nx CKD model mice with decreased physical performance, after these mice were treated with ALA for 8 weeks. The results suggest that ALA-treatment can contribute to the improvement of physical performance and glucose tolerance of the pathological conditions where muscle mitochondrial functions are reduced.

It is known that ALA serves as a precursor for biosynthesis of heme and cytochrome c, and ALA-treatment increases these electron carriers in mitochondria and hence activates mitochondrial electron transport system and enhances ATP production^18^. In cultured cells, 100 μM of ALA with SFC demonstrated a nearly 10-fold increase in heme^28^. In the present study, ALA was shown to increase expression of PGC1α, the master regulator of mitochondrial biogenesis, and enhanced muscle mitochondria. However, the increase in mitochondria after ALA administration was not observed in all organs examined. Therefore, it was suggested that the biological processes affected by administration of ALA, including ALA uptake, formation of heme and cytochrome c, and the effects on mitochondrial electron transport, would vary from one organ to another.

In cultured myocytes, ALA increased the expression of PGC1α and mitochondrial amount and elevated the oxygen consumption rate. On the other hand, when the cells were treated with antimycin A, an inhibitor of mitochondrial electron transport system, the increases in the expression of PGC1α, mitochondrial amount and oxygen consumption rate by ALA were abrogated. The findings suggest that ALA-induced promotion of mitochondrial electron transport itself serves as a signal to promote mitochondrial biogenesis and oxygen utilization. In addition, ALA-induced decrease in the expressions of genes modulating muscle mass, BCAT1 and atrogin1, was also abolished by antimycin A. This fact implies that the promotion of mitochondrial electron transport in the myocytes by ALA can affect muscle mass. Our current finding is compatible with the previous study which showed that muscle mass was deceased in the mouse model of mitochondrial dysfunction^16^.

It should be emphasized that muscle mass was significantly increased in ALA-treated mice, which was associated with an increase in BCAAs contents, which consisted of isoleucine, leucine and valine. BCAAs are known to stimulate muscle protein synthesis^29^. BCAAs promote muscle synthesis through activation of the mammalian target of the rapamycin (mTOR) and S6K1 system and suppress muscle degradation through inhibiting the formation of proteolytic autophagosomes by reducing the expression of atrogin1 and MuRF1. In a previous report, leucine treatment in rats significantly increased the lean body mass when compared to control rats (the percentage of lean mass: 90.1% ± 0.6% vs 92.3% ± 0.8%, p = 0.04)^30^. Leucine-enriched essential amino acid mixtures in elderly people for 8 months were effective to significantly enhance muscle mass in legs, arms and the body trunk^31^. In BCAT-knockout mice with increased levels of muscle BCAAs, the activity of mTOR signaling and protein synthesis rate were increased, and the insulin sensitivity was enhanced^32^. In elderly people, fasting plasma BCAAs were significantly lower than healthy young people^33^. Based on these facts, BCAA supplements are expected to be useful for sarcopenia treatment.

In the metabolome analysis of the quadriceps muscle of ALA-treated mice, all three BCAAs: isoleucine, leucine, and valine, were increased, while the total level of 20 amino acids tended to decrease. Because BCAAs are essential amino acids and thus enhanced biosynthesis can be ruled out, the suppression of BCAA degradation by ALA was suggested. Indeed, the quantitative PCR analysis showed that the expression levels of BCATs, which are enzymes that catalyze the first step of BCAA degradation, were markedly reduced in the quadriceps muscle of ALA-treated mice. Therefore, ALA may increase BCAAs through decreased expression of BCATs. In cultured myocytes, the BCAT expression was significantly decreased by ALA and the ALA-induced decrease in BCAT expression disappeared under the treatment with antimycin A, an inhibitor of the complex III in the mitochondrial electron transport chain. Therefore, the promotion of mitochondrial electron transport by ALA was suggested to serve as a stimulus to decrease BCAT expression. These findings indicate that ALA is useful for improving muscle mass, physical performance and glucose tolerance in elderly sarcopenic patients by activating skeletal muscle mitochondria and increasing BCAAs. In the previous study, we examined the effects of a mitochondrial electron carrier, coenzyme Q10 (CoQ10) on the physical performance of mice. Administration of CoQ10 in mice with decreased muscle mitochondrial activity, enhanced mitochondria and improved exercise endurance but did not increase muscle mass^17^. We speculate that bioactive substances that are derived from ALA and subsequent porphyrin synthesis pathway, which include other molecules than the mitochondrial electron carriers, heme and cytochrome c, might also contribute to muscle enhancement by ALA. The precise mechanism by which ALA regulates BCAT expression and muscle mass should be further examined.

As mentioned above, BCAAs would be useful for the treatment of sarcopenia, however, recent studies demonstrated that direct administration of BCAAs had a risk to cause insulin resistance^34^. Increased levels of serum BCAAs were positively correlated with the homeostasis model assessment (HOMA) index, which is an index of insulin resistance^35^. BCAAs have been shown to inactivate insulin receptor substrate 1 (IRS-1) through S6K1-dependent serine phosphorylation of IRS-1^36, 37^. On the other hand, the blood glucose reducing effect of ALA has been reported in various animal and human studies. In obese diabetic rats, ALA has been shown to improve glucose tolerance^38^. In double-blind interventional studies in patients with impaired glucose tolerance, ALA has been shown to improve fasting blood glucose level and HbA1c^39–41^. In the present study, even though ALA-treatment might promote eating behavior and thus gain body weight, the blood glucose levels reduced in a wide spectrum of disease models of mice, including obesity, aging and chronic kidney disease. Therefore we believe that ALA-treatment would be applicable for various types of diabetes mellitus with sarcopenia in future therapies.

A previous report has shown that mitochondrial dysfunction in the skeletal muscle exacerbates blood glucose levels^42^. In addition, a reduction in muscle mass is also associated with insulin resistance^43^. Therefore, we believe that the enhancement of mitochondrial function and muscle mass by ALA-treatment together improved glucose tolerance. In ALA-treated mice in which food intake was matched with untreated mice by a pair-feeding method, the oxygen utilization significantly increased and body weight decreased. Based on a report showing that appetite is increased by activating hypothalamic mitochondria^44^, there is a possibility that ALA might pass across the blood brain barrier and stimulate the appetite center to increase appetite by activating mitochondria. In obese db/db mice with markedly elevated blood glucose and urine volume, a suppression of osmotic diuresis due to ALA-induced glucose lowering effect might contribute to an increase in body weight associated with ALA treatment. From these facts, caution should be exercised for possible weight gain due to increased food intake when ALA is to be administered to diabetic patients with obesity. Meanwhile, diabetic patients with sarcopenia due to reduced food intake and consequent malnutrition would be suitable candidates for ALA treatment.

**Figure 6:**
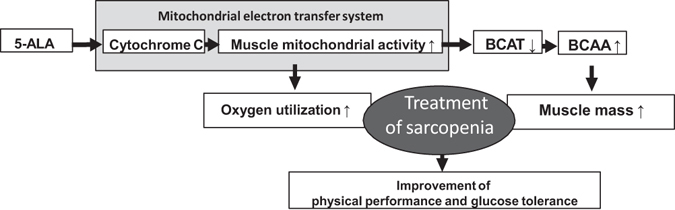
Schematic representation of the results of the present study. We found that ALA improved physical performance and glucose tolerance by increasing muscle mitochondria and promoting oxygen utilization as well as by increasing BCAAs and muscle mass through suppression of BCATs expression. 5-ALA, 5-aminolevulinic acid; BCAT, branched-chain amino acid aminotransferase; BCAA, branched chain amino acid.

In the present study using ALA-treated mice, we found that ALA improved physical performance and glucose tolerance by increasing muscle mitochondria and promoting oxygen utilization, as well as by increasing BCAAs and muscle mass through suppression of BCAT expression (summarized in Fig. 6). Sarcopenia not only shortens healthy life expectancy due to deterioration in physical performance but also worsens survival rate due to increases in metabolic diseases and cardiovascular diseases. Mitochondrial activation with ALA is expected to contribute to true sarcopenia treatment that not only enhances muscle mass and physical performance but also ameliorates glucose tolerance and brings about an improved cardiovascular prognosis.

## Electronic supplementary material

Supplementary Information
