# Supplementary material for: Treatment of sarcopenia and glucose intolerance through mitochondrial activation by 5-aminolevulinic acid
Source: Sci Rep. 2017 Jun 21;7:4013. doi: 10.1038/s41598-017-03917-0 (PMC5479778; doi:10.1038/s41598-017-03917-0)

Supplementary Information

Treatment of sarcopenia and glucose intolerance through mitochondrial activation by 5-aminolevulinic acid

Chikako Fujii, Kazutoshi Miyashita, Masanori Mitsuishi, Masaaki Sato, Kentaro Fujii, Hiroyuki Inoue, Aika Hagiwara, Sho Endo, Asuka Uto, Masaki Ryuzaki, Motowo Nakajima, Tohru Tanaka, Masanori Tamaki, Ayako Muraki, Toshihide Kawai and Hiroshi Itoh

The full-length blots for P-S6K1 and total S6K1 of Figure 1O.

Western blots of P-S6K1 and total S6K1 in the quadriceps muscle of ALA-treated mice. p70 S6 kinase means S6K1.

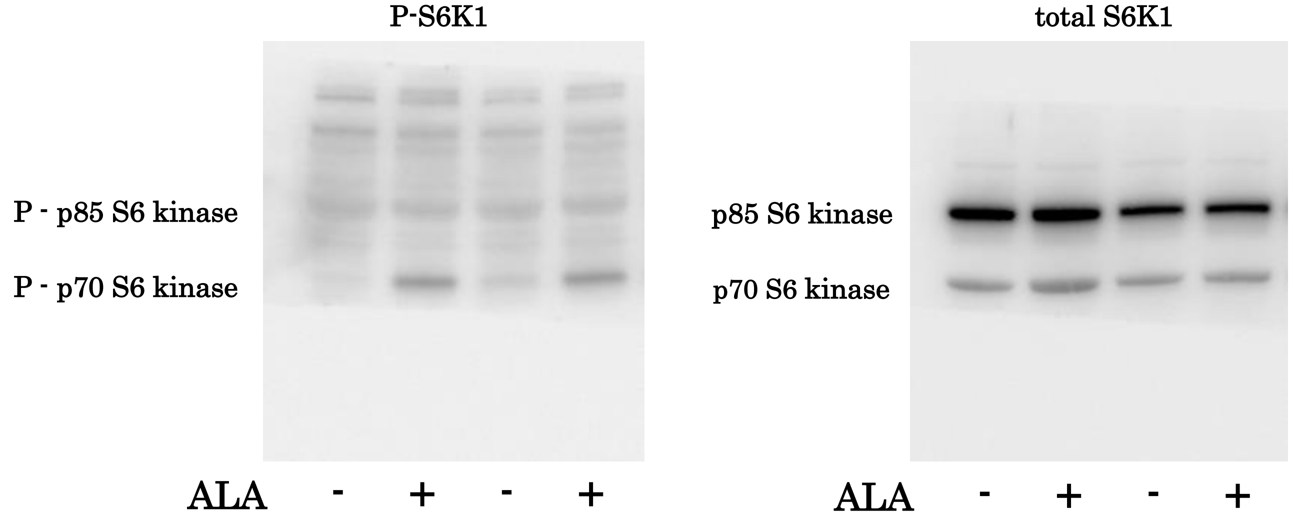

Supplement: Supplementary file 1 — Supplementary Information [file 41598_2017_3917_MOESM1_ESM.pdf]
